# Normative Values of Retinal Nerve Fibre Layer Thickness and Optic Nerve Head Parameters and Their Association with Visual Function in an African Population

**DOI:** 10.1155/2020/7150673

**Published:** 2020-02-11

**Authors:** Stephen Ocansey, Emmanuel Kwasi Abu, Andrew Owusu-Ansah, Shadrack Mensah, John Oduro-Boateng, Rene Abalo Kojo, Samuel Kyei, Samuel Bert Boadi-Kusi, Obed Amoah-Smith, Enyam Komla Amewuho Morny, Charles Darko-Takyi, Carl Halladay Abraham, Benjamin Appiah Nyamekye, Alex Azuka Ilechie

**Affiliations:** ^1^Department of Optometry, School of Allied Health Sciences, College of Health and Allied Sciences, University of Cape Coast, Cape Coast, Ghana; ^2^Glaucoma Research Group, 2nd Xiangya Hospital of Central South University, Changsha, China; ^3^Eye Unit, Nsawam Government Hospital, Nsawam, Eastern Region, Ghana; ^4^Emmanuel Eye Medical Centre, Adamfio Link East Legon, Accra, Ghana; ^5^Eye Unit, Directorate of University Health Services, University of Cape Coast Hospital, Cape Coast, Ghana

## Abstract

**Purpose:**

To determine normative values of retinal nerve fibre layer thickness (RNFL) and optic nerve head (ONH) parameters and their association with routine clinical tests such as refractive error (RE), stereoacuity (SA), and contrast sensitivity (CS) in an African population.

**Methods:**

In a cross-sectional observational study, 100 normal subjects aged 20 to 78 years were evaluated using the Cirrus HD-OCT 5000 and matched with 200 glaucoma patients.

**Results:**

Average (±SD) RNFL thickness for normal subjects was found to be 102.37 ± 7.45 (range, 82–119 microns) compared with 90.74 ± 14.50 found for glaucoma subjects. Females had higher average RNFL values (104.84 ± 6.90) compared with males (99.80 ± 7.18). Significant associations were calculated between quadrant RNFL thickness and SA, SE, and CS (all *p* < 0.05). The mean cup to disc ratio (CDR) was 0.49 ± 0.12, and mean optic disc area (DA) was 2.08 mm^2^ ± 0.40. Smaller DA was recorded for participants aged 60+ years (1.86 ± 0.25), followed by 40–59 age group (2.01 ± 0.41) and then 20–39 age group (2.19 ± 0.41). Significant associations were calculated between SA and ONH parameters, except rim area (all *p* < 0.05). The mean cup to disc ratio (CDR) was 0.49 ± 0.12, and mean optic disc area (DA) was 2.08 mm^2^ ± 0.40. Smaller DA was recorded for participants aged 60+ years (1.86 ± 0.25), followed by 40–59 age group (2.01 ± 0.41) and then 20–39 age group (2.19 ± 0.41). Significant associations were calculated between SA and ONH parameters, except rim area (all *p* < 0.05). The mean cup to disc ratio (CDR) was 0.49 ± 0.12, and mean optic disc area (DA) was 2.08 mm^2^ ± 0.40. Smaller DA was recorded for participants aged 60+ years (1.86 ± 0.25), followed by 40–59 age group (2.01 ± 0.41) and then 20–39 age group (2.19 ± 0.41). Significant associations were calculated between SA and ONH parameters, except rim area (all

**Conclusion:**

RNFL thickness in healthy black Ghanaian population was significantly higher than that reported in other races. The values and associations reported in this study can inform clinical decision on the normal variation in RNFL and optic disc parameters.

## 1. Background

Glaucoma is an asymptomatic ocular disease characterized by a progressive loss of retinal nerve fibres and increased cupping of the optic disc due to several cellular disease phenomena in the eye [1]. Glaucoma, particularly primary open-angle glaucoma (POAG), is the leading cause of irreversible blindness in the world [2, 3]. Global projections indicate that, by 2020, about 79.6 million people will have the disease and 11.2 million cases will go blind, making the disease a major public health concern [2, 4]. In particular, individuals of African descent have an increased prevalence to POAG and have poorer visual outcomes indicating additional genetic risk factor of originating in Africa [5–7]. Blacks are 4.3 times more likely to have glaucoma than other races [7]. POAG is known to account for 15% of blind cases annually in Sub-Saharan Africa. In Ghana, a population-based study showed that the prevalence of POAG was 8.5% in persons 40 years and above, contributing to around 20% of the burden of blindness, making Ghana the highest prevalent country in Africa and second in the world [8–10].

Traditionally, high intraocular pressure (IOP) (greater than 21 mmHg) is regarded as the main risk factor for glaucoma, so medical diagnosis in low-resource environments is based on measuring IOP and monitoring the appearance of the optic disc. However, recent studies have shown that glaucoma may develop even with normal levels of IOP and substantial number of people with high IOP do not develop glaucoma [11]. These new findings make use of IOP as a main marker to detect inadequate and defective glaucoma. Confirmatory glaucoma tests such as visual fields (VFs) are often expensive and not readily available to patients. In addition, thinning of the retinal nerve ﬁber layer (RNFL) can occur with little or no noticeable functional changes in the early stages of glaucoma, and therefore early detection using VF tests becomes difficult. It is reported that up to 50% of retinal ganglion cells (RGCs) are lost before any functional deficits are seen on VFT [5, 12, 13].

Optical coherence tomography (OCT), a modern diagnostic imaging technique, is increasingly being utilized as an important imaging modality in the evaluation and management of retinal diseases, particularly POAG as it is able to detect early structural changes in the retinal nerve fibre layer (RNFL) and optic nerve head (ONH). The emergence of this noninvasive test and its ability to image (scan) intraocular structures in vivo with resolution approaching that of histologic sections has made OCT particularly useful in the detection and quantification of POAG in low-resource settings like Ghana, where majority of patients are treated medically [8, 11, 14].

The OCT, first described by Huang et al. [14], gathers information for diagnosing glaucoma and retinal disorders by comparing data it generates with population-derived normative values. Research has however established a strong racial and ocular variation in RNFL thickness and ONH parameters [15–19]. Population-derived normative measures of ONH and RNFL are therefore important to quantify deviations from normal ranges [20–22]. There is limited preexisting normative values for an African population regarding the differences in RNFL thickness and ONH parameters measured with spectral domain OCT (SD-OCT). The SD-OCT collects data 100 times faster than conventional time-domain (TD) OCT [11, 23]. To the best of our knowledge, only two studies by Mashige and Oduntan [24] in black South Africans which used iVue-100 SD-OCT and Sani et al. [25] in Nigeria which used Stratus TD-OCT can be cited. Though Ghana is known to have the highest prevalence of glaucoma cases in West Africa, no normative data for the Ghanaian population have been determined, and therefore judgment on the integrity of RNFL and OHN parameters is made with reference to other races. Normative data for the Cirrus SD-OCT were generated in a study that included 284 participants, with a sample of 51 African Americans comprising 35% of the study population [20]. This makes current application of established normative data for the African populations not extensively precise, which may lead to overestimation or underestimation resulting in misdiagnosis. This study, being the first attempt to document normative values for OCT imaging in Ghana, seeks to generate normative values for RNFL thickness and ONH parameters in the Ghanaian population using Cirrus SD-OCT and establish a relationship between RNFL and ONH parameters and some demographic and ocular parameters. Knowledge of the differences in the physiological variations of the ONH and RNFL is important to quantify as they may affect the ability to detect glaucoma and determine the normative ranges for OCT devices in clinical practice in specific racial groups.

## 2. Methods

### 2.1. Setting

Ghana is a sub-Saharan West African country with a population of 28.4 million, a total area of 238,533 km^2^ (land: 227,533 km^2^ and water: 11,000 km^2^), and divided into 16 administrative regions [9]. There are 216 districts, which form the basic health units in Ghana. There is an emerging elderly population due to recent marked increase in life expectancy. Life expectancy at birth is 57 years for males and 61 years for females. Due to the high acquisition and operating cost, only a few specialist eye centers in Ghana provide SD-OCT scan services. This study was, therefore, conducted at a specialist center, Emmanuel Eye Medical Centre, in Ghana's capital city, in the Greater Accra. The regions multiethnicity (major ethnic groups in Ghana are Akans, Akwapims, Ewes, Akims, Northern tribes, and Ga-Adangbe) made it a suitable area for this study (conﬁrmed by interviews with participants). The center which also has specialist glaucoma ophthalmologists is a known national referral facility for glaucoma patients.

### 2.2. Study Design

A hospital-based observational, cross-sectional study was conducted to provide normative values for RNFL thickness and ONH parameters by adhering to the tenets of the Declaration of Helsinki on Research Involving Human Subjects. The study received approval from the Institutional Review Board of the University of Cape Coast and from the management of Emmanuel Eye Medical Centre, Accra. The study was a noninvasive one; however, care was taken to lessen minimal risks such as eye tiredness and brief exposure of the eye to instruments. Participants provided written informed consent after being verbally informed that their participation was purely on a voluntary basis.

### 2.3. Study Population and Participant Selection

A total of 100 nonglaucoma subjects between the ages of 20 and 78 years were recruited for the study based on the resources available. They were recruited from the friends and family members of patients, trainees, and staff at the study center. Participants' enrollment was purely voluntary, after explanation of the nature and possible consequences of the study, and was standardised to ensure adequate age and sex. Participants were grouped as follows: 19–39 years, 40–59 years, and 60 years and above and compared with 200 glaucomatous patients in ratio of 1 : 2. The control cohort of glaucoma patients was selected from a clinical database from the Emmanuel Eye Medical Centre or subjects were enrolled prospectively. They were all patients diagnosed or previously diagnosed by an ophthalmologist as having glaucoma.

### 2.4. Inclusion and Exclusion Criteria

The purpose of this study was to establish baseline data, and as such, a number of strict inclusion and exclusion criteria were applied to recruit participants with normal eyes. Each subject underwent a detailed medical history and ophthalmic examination to determine the eligibility by a team of eye care personnel comprising an ophthalmologist (from Emmanuel Eye Medical Centre) and optometrists from the Department of Optometry and Vision Science, University of Cape Coast. Eligibility for inclusion comprised the following: no known eye disease (pathology), no visual impairment (best corrected visual acuity worse than 0.48), IOP below 20 mmHg, and no obvious retinal disease or defect. Visual fields were checked to ensure those included had normal visual field findings in both eyes defined by a mean deviation and pattern standard deviation within 95% confidence limits, a glaucoma hemifield test result within normal limits, as determined by both the consulting ophthalmologist and visual field technician. A reliable test result was defined as fixation loss rates of 15% or less, false-negative responses, false-positive responses, and no visual field loss consistent with ocular or systemic disease. Subjects with a history of ocular diseases or pathology with residual visual impairment, retinal diseases, and refractive error greater than −6.00 DS and +6.00 DS were excluded from the study. Subjects with an average cup to disc ratio of 0.5 and above, amblyopia, and history of intraocular surgery or laser therapy were also excluded. Subjects were also ruled out if they had elevated intraocular pressure of 20 mmHg or greater, RNFL thickness of less than 80 *µ*m, optic cup/disc ratio of 0.5 or greater, and average cup volume of 0.30 cm^3^ or greater. Participants with glaucoma hemifield test results outside the normal limit and pattern standard deviation with probability value of <5% were also excluded.

### 2.5. Data Collection Procedure

Individuals who volunteered to take part in the study signed informed consent and subsequently underwent ophthalmic examinations consisting of general ocular health assessment using Haag Streit slit lamp biomicroscopy, fundus examination with Welch Allyn ophthalmoscopes, IOP measurement using the Goldmann applanation tonometer, objective autorefraction (Humphrey Zeiss 599), and visual field quantification with the Swedish interactive threshold algorithm (SITA) standard 24 to 2 Humphrey Visual Field Analyzer (Carl Zeiss Meditec, Inc.) program. Then, those who satisfied the inclusion criteria underwent clinical visual test assessments consisting of distance visual acuity (VA) measurement using Snellen charts, contrast sensitivity (CS) measured with the Pelli Robson CS chart, and stereoacuity with the TNO stereo chart. Subsequently, RNFL and ONH parameters were assessed using the Cirrus HD-OCT 500 model (Carl Zeiss Meditec, Dublin, CA, USA). OCT scans were taken by a trained ophthalmic technician, who was required to take all scans used in this study. Optimal pupillary alignment, fundus focus, illumination, and centration of the optic disc were ensured before each scan was taken. After pharmacologic dilatation, both eyes were scanned 5 times each using the optic disc cube 200 × 200 protocol. The detailed procedure for the Cirrus HD-5000 OCT imaging has been described elsewhere [20, 22].

**Figure 1:**
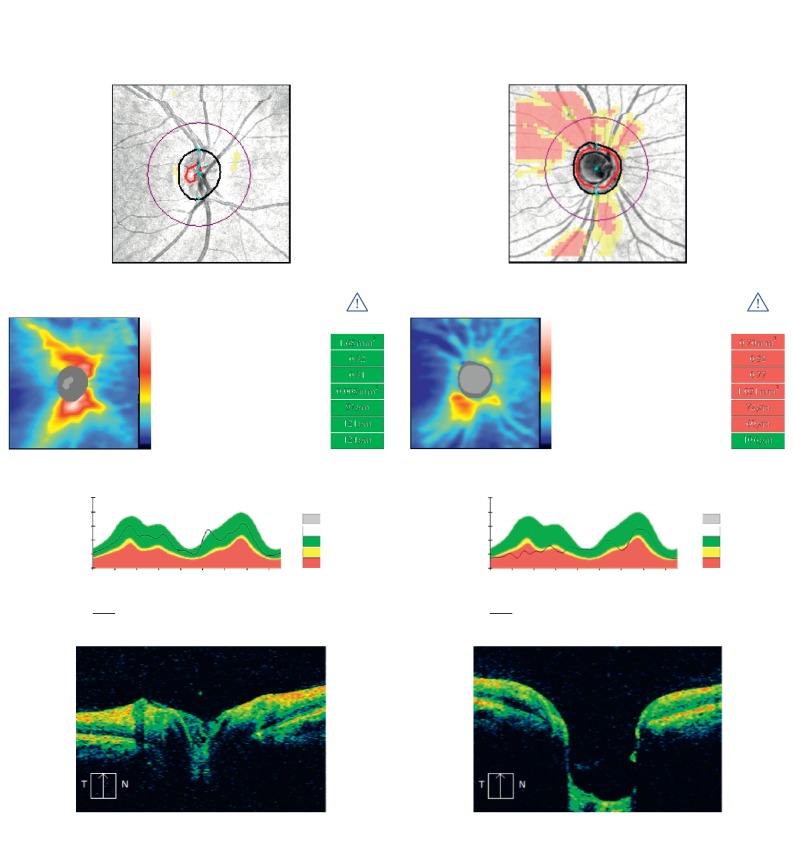
Cirrus HD-OCT 5000 scans of the right eye (OD) normal and glaucomatous patients, showing ONH and RNFL parameters. RNFL and ONH parameters within normality are colour coded green, values below normality are colour coded red, and borderline values are colour coded yellow. White colour code refers to values above normality range, and grey indicates normative data are not applicable.

The following variables were considered in this study. Visual acuity of both eyes, age, gender, IOPs of the right and left eyes in mmHg, spherical equivalent of refractive error, contrast sensitivity, stereoacuity; RNFL parameters: average, superior, inferior, temporal, and nasal RNFL; and ONH parameters: rim area (RA), cup volume (CV), disc area (DA), average cup disc ratio (ACDR), and vertical cup disc ratio (VCDR). The CDR is calculated by taking the ratio of the area of the “cup” portion of the optic disc with the area of the optic disc. The VCDR is the ratio of the cup diameter to the disc diameter in a vertical meridian through the cup center. The CV is a 3-dimensional measurement defined as the volume between the plane created at 200 *μ*m offset to the plane of the disc and the vitreoretinal interface. By default, the right eye from each enrolled subject was chosen for inclusion in the normative database. For the purpose of this study, only HD-OCT scans with signal strength of 9/10 and above with no saccades in the enface image were deemed as normal and used for analysis. Copies of data files were stored in the machine as backup.  Figure 1 shows Cirrus SD-OCT scans of normal and glaucomatous right eyes.

### 2.6. Data Analysis

Data were entered into Statistical Package for Social Science (SPSS) version 25.0 for macOS and analysed. Descriptive statistics used in the study were means, standard deviations, percentages, and frequencies of variables. The relationship between subject demographic and ocular characteristics including age, sex, stereoacuity, contrast sensitivity, and mean refractive spherical equivalent and measured RNFL and OHN parameters were compared using 1-way analysis of variance (ANOVA) with 95% confidence intervals. Correlations between ONH and RNFL parameters as well as age and RE were assessed with Pearson and Spearman correlation coefficients. Associations between age and ONH and RNFL parameters were evaluated using general linear models (GLMs). ANOVA analysis was used to compare glaucoma and normal and binary logistic regression used to test associations after adjusting for age. The alpha level was for significant associations set at *p*=0.05, thus an association would exist when *p* ≤ 0.05.

## 3. Results

### 3.1. Demographics and Ocular Characteristics

The analyses considered 100 subjects who were included in the normative database. Out of the total 100 nonglaucoma subjects enrolled in the study, 49 were men (49.0%) and 51 were women (51.0%). Their mean age was 40.6 years (standard deviation (SD), 14.5; range, 20–78).

The demographic and clinical visual function variables of the 100 subjects included in the analysis are shown in Table 1. Mean visual acuity was 0.30 (SD, 0.34) LogMAR (corresponding to Snellen 6/12). Spherical equivalent (SE) of the refractive errors ranged from −5.25 to + 4.50 dioptres (D), with a mean refractive error of −0.50 (SD, 1.5). Mean contrast sensitivity was 10.40 triplets (SD, 1.20; range, 5–13 triplets), mean stereoacuity value was 67.65 arc sec (SD, 9.71; range, 15–480 arc sec), and mean intraocular pressure of all participants was 14.79 mmHg (SD, 2.60; range, 10–20). A 1-way analysis of variance followed by post hoc least significant difference for tests for age revealed that, for stereoacuity, contrast sensitivity, IOP, and visual acuity, there was a statistically significant variation (<0.001) between the age groups 19–39 and 60+; then, the age group 40–59 also showed significant variation from age group 60+. There was however no significance differences between gender for all tests measured (Table 1).

### 3.2. RNFL Parameter Measurements and Demographic/Visual Parameters

**Figure 2:**
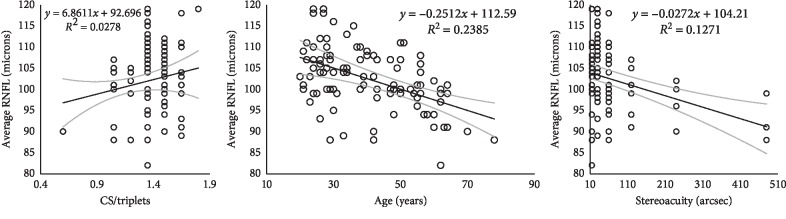
Scatter plots showing the relationship between age (years), stereoacuity (sec arc), contrast sensitivity (no. of triplets read on a Pelli Robson CS chart), and average retinal nerve fibre layer thickness (AVG_RNFL) (*μ*m). In the first and outer panels, it shows a decrease in RNFL thickness with age and stereo thresholds, respectively, with a decrease in RNFL thickness.

The distribution of RNFL thickness as measured by Cirrus SD HD-OCT in the 100 normative database subjects is shown in Table 2. The mean RNFL thickness recorded for the study sample was 102.37 ± 7.45 (range, 82–119 microns), with female participants having higher average RNFL values −104.84 ± 6.90 compared with that of males, 99.80 ± 7.18. Inferior quadrant RNFL had the highest thickness value of 133.60 ± 13.84 (range, 87–161 microns) followed by superior RNFL 131.54 ± 14.79 (range, 104–68 microns), nasal RNFL 76.73 ± 12.6 (50–116 microns), and temporal RNFL 66.83 ± 9.48 (range, 50–104 microns). RNFL thickness was observed to vary with age (Figure 2), with statistically significant differences (*p* < 0.001) between the age groups (1-way analysis of variance). Participants aged between 20 and 39 years recorded the highest average RNFL thickness of 105.20 ± 7.10 microns followed by the 40–59 year group with 101.50 ± 5.76 microns and then those aged 60 years and above with 94.80 ± 6.58 microns.

### 3.3. ONH Parameter Measurements and Demographic/Visual Variable

Table 3 shows the distribution of ONH parameters as measured by Cirrus SD HD-OCT in the 100 normative database subjects. The mean (SD) cup to disc ratio (CDR) recorded was 0.50 ± 0.12 (SD, 0.13; range, 0.09–0.70), and mean vertical optic cup to disc ratio (VCDR) was 0.47 (SD, 0.13, range, 0.09–0.70) with females having slightly larger CDR (0.50 ± 0.14) compared with males (0.49 ± 0.12). The mean (SD) optic disc area (DA) was 2.08 mm^2^ (SD, 0.40; range, 0.90–3.09) with females having slightly larger disc areas (2.11 ± 0.40) than males (2.04 ± 0.41). Other ONH measures were as follows: mean optic rim area (RA) was 1.48 mm^2^ (SD, 0.21; range, 0.89–2.23) and mean optic cup volume was (CV) 0.19 mm^3^ (SD, 0.14; range, 0–0.67) with females having slightly higher values. Smaller disc areas were recorded for participants aged 60+ years (1.86 ± 0.25), followed by those in the 40–59 year group (2.01 ± 0.41) and then 20–39-year group (2.19 ± 0.41). ANOVA calculated revealed that no statistical difference between gender, refractive error, and age (except age and RA and DA; RE and DA; all *p* < 0.05).

### 3.4. Association between ONH and RNFL Parameters and Clinical Variables

A bivariate correlation statistics calculated the strength of the linear relationship between RNFL thickness and demographics and clinical variables (Table 4). The Pearson correlation showed a statistically significant association between all RNFL quadrants and age, except temporal RNFL (all *p* < 0.01) which showed a moderate negative correlation (all *r*^2^ > 27% < 50%). Sex also showed significant relationship with RNFL parameters, except temporal quadrant RNFL thickness (all *p* < 0.05). With regards to measured visual function parameters, spherical equivalent refractive error showed a significant association with temporal RNFL thickness (*p* < 0.05) with weak negative correlation (*r*^2^ = 23%). Stereoacuity showed a significant association with average RNFL thickness (*p* < 0.05) with a negative correlation (*r*^2^ = 27%). Contrast sensitivity showed a significant relation with nasal RNFL quadrant thickness (*p* < 0.05) but with weak correlation (*r*^2^ = 22%). IOP and visual acuity did not show any significant correlation with RNFL quadrant thickness. Figure 2 shows the relationship between average RNFL thickness and age, stereoacuity (sec arc), and contrast sensitivity (triplets).

In a generalised linear model (GLM) (supplementary [Supplementary-material supplementary-material-1]), only age (*p*=0.006) and sex (*p*=0.017) had significant effect on average RNFL thickness, ((*F*(3, 96) = 13.8), *p* < 0.001, *R*^2^ = 0.24) demonstrating a 0.25 *μ*m decrease in RNFL thickness per yearly increase in age. The parameter estimates for RNFL thickness show an increase of 4.42 *μ*m for those aged 19–30 and 2.67 *μ*m increase for those aged 40–59 over those 60+ years per yearly decrease in age. Females showed 5.5 *μ*m increase in average RNFL thickness compared with males (Supplementary [Supplementary-material supplementary-material-1]).

Pearson correlation statistics revealed a significant association between visual acuity and ONH parameters, except rim area (all *p* < 0.01). There was a moderate correlation between VA and ONH parameters (all *r*^2^ > 26% < 31%). Spherical equivalent refractive error showed a significant association with disc area and average CDR (all *p* < 0.05), with weak positive correlation (all *r*^2^ > 20% < 22%). Age showed significant relation with rim area and disc area (all *p* < 0.01) with a moderately weak negative correlation (all *r*^2^ > 29% < 31%). Sex, IOP, contrast sensitivity, and stereoacuity did not show any significant correlation.

**Figure 3:**
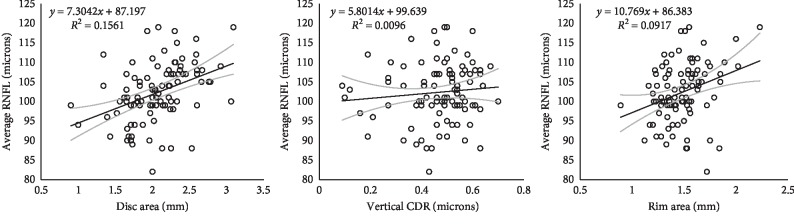
Relationship between disc area (mm^2^), vertical cup-disc ratio (microns), rim area (mm^2^), and average retinal nerve fibre layer thickness (AVG_RNFL) (*μ*m), showing an increase in RNFL thickness with increasing optic disc area.

Pearson correlations were also run to investigate the association between RNFL parameters and ONH parameters. As indicated in Table 4, DA showed a significant association with nearly all RNFL parameters, with the exception of the temporal and nasal RNFL quadrants (*p* < 0.001), with moderate positive correlation between DA area and RNFL parameters (all *r*^2^ > 30% < 40%). DA showed a significant association with other ONH parameters (all *p* < 0.001) with moderately strong positive correlation (*r*^2^ > 49%). Rim area showed a similar association pattern, with a significant association between average RNFL, superior RNFL, and inferior RNFL (all *p* < 0.008). Correlation was positively weak to moderate (*r*^2^ > 25% < 31%). Rim area showed a significant association with DA, vertical CDR, and cup volume (all *p* < 0.05). Rim area was positively correlated with disc area (*r*^2^ = 49%); however, RA showed a weak negative correlation with VCDR and cup volume (*r*^2^ < 25%). The ACDR showed a significant association with average and nasal RNFL (*p* < 0.05) with a weak positive correlation (*r*^2^ = 20.8%). Cup volume showed a significant association with nasal RNFL (*p* < 0.05) and a weak positive correlation (*r*^2^ = 22.3%). Cup volume showed a significant association with other ONH parameters (all *p* < 0.01) with a strong positive correlation with disc area, VCDR, and ACDR (*r* > 50%). It however showed a weak negative correlation with rim area (*r*^2^ < 25%).  Figure 3 shows the relationship between average RNFL thickness and ONH parameters, depicting an increase in average RNFL thickness with increasing optic disc area.

### 3.5. RNFL and ONH Variables in Normal and Glaucomatous Eyes

Table 5 shows a comparison between RNFL and ONH parameters in normal eyes and glaucoma cases currently under the treatment taken with the Cirrus HD-OCT 5000. The mean (SD) RNFL value for the glaucomatous subjects was 90.74 ± 14.55 (*μ*m), compared with 102.37 ± 7.45 (*μ*m), recorded for the normative data set, showing a 11.4% reduction in RNFL thickness. Similar trends in reduction were observed for the various RNFL quadrant thicknesses. There were statistically significant differences (*p* < 0.001) between normal and all glaucomatous RNFL and ONH parameters. For each RNFL quadrant thickness, it was observed that there was generally between 11–22% reduction in RNFL thickness. For ONH parameters, comparisons between normal and all glaucomatous eyes yielded statistically significant differences (*p* < 0.001) for all parameters measured. From Table 5, it can be observed that there are significant increases in both cup volume, vertical cup to disc ratio, and average disc ratio values in the glaucoma data compared with normative data acquired in this study. Binary logistic regression analysis with age correction revealed that a yearly increase in age increases the likelihood of getting glaucoma by 1.19 times (Supplementary [Supplementary-material supplementary-material-1]).

## 4. Discussion

Glaucoma is the leading cause of blindness among blacks, especially among individuals of African background. Individual risk factors go beyond socioeconomic factors such as levels of education, income, and family history, extending to genetics and the environment. It is important, therefore, that racial background should be considered in designing diagnosis protocols for glaucoma. This study derived the normative data on RNFL thickness measurements and ONH parameters using the Cirrus HD-OCT 5000 and its association with clinical ocular measurements.

### 4.1. Normative Data

The average (SD) RNFL thickness, 102.37 ± 7.45 *μ*m, found in this study is considerably higher than average reported using the Cirrus HD-OCT. Knight et al. [20] who presented data on 284 normal multiethnic (European, Chinese, African, and Hispanic) subjects aged 18–84 years reported a smaller average RNFL thickness of 94.0 *μ*m (SE 0.6) (adjusted for age and disc area), and Tariq et al. [22] found 99.4 ± 9.7 *μ*m in an East Asian and white populations. The present study calculated similar values to those in studies conducted on black Africans. Similar studies carried out on African populations in Nigeria and South Africa reported averages of RNFL 104.2 ± 10.7 *μ*m and 110 ± 7.4 *μ*m, respectively [24, 25] (Table 6). There is a noticeable +8 *μ*m difference in the average RNFL thickness, particularly due to differences in nasal and temporal quadrants, while data on a Nigerian population showed a difference of +2 *μ*m in average RNFL. It can be observed that normative data for Asian subjects [19, 28] showed identical mean RNFL thickness; however, we recorded higher superior and inferior RNFL quadrant thickness but lower nasal and temporal RNFL quadrant thickness. Cirrus OCT normative average RNFL values published for whites are 90.1 *μ*m (European descent) and 95.6 *μ*m (Hispanic descent) [20], which are significantly low compared with values obtained for the African population confirming racial variation in RNFL thickness. Other published data on Caucasians [21, 22] revealed similar lower RNFL thickness values.

Consistent with published data (Table 6), RNFL was thickest in the inferior quadrant, followed by the superior quadrant, nasal quadrant, and temporal quadrant [20, 22, 24] and followed the ISNT rule. Age was observed to have a negative correlation with all RNFL parameters (Figure 1). RNFL was observed to decline gradually as age increases with a 0.25 *μ*m reduction in mean RNFL thickness per year of aging. Similar published finding of 0.38 *μ*m per year has been reported using scanning laser polarimetry [29] and OCTs by Knight and colleagues [20], 0.19 *μ*m/y, and Mashige and Oduntan [24] at a rate of decline of 0.11 *μ*m/y [24]. Sex was found to affect all retinal nerve layers with females, 104.84 ± 6.90, having higher RNFL thickness compared with males, 99.80 ± 7.18. This study however found only small sex differences in RNFL thickness, similar to ﬁndings from other studies [21, 30, 31]. On the other hand, ONH parameters found in this study had similar values compared to published normative values for Cirrus HD-OCT 5000 [20]. The mean disc area was 2.0 mm^2^ comparable to 1.9 mm^2^ found by Knight et al. [20], 1.83 ± 0.35 mm^2^ by Mwanza et al. [32], and 1.98 ± 0.38 mm^2^ by Tariq et al. [22] all using Cirrus OCT but smaller than 2.34 ± 0.41 2.47 mm^2^ found by Girkin and colleagues [33] and 2.49 mm^2^ by Marsh et al. [34] for individuals of African descent (AD) all using Stratus OCT. Normative values between 2.34 ± 0.41^2^ and 2.63 ± 0.55 mm^2^ have been recorded for the Stratus OCT [31, 35, 36]. Previous studies have reported smaller DA for individuals of Chinese (1.9 mm^2^ (AD) vs. 1.8 mm^2^), European descent (−1.7 mm^2^), and Hispanics (1.9 mm^2^) using the Cirrus OCT [20] and Stratus OCT for European descent (2.49 mm^2^ vs. 2.17 mm^2^) and Hispanic individuals (2.33 mm^2^) [34].

Population studies that measured normal disc size using Stratus have reported cup-disc area ratios between 0.17 ± 0.11 and 0.37 ± 0.20, which are substantially smaller than our ACDR and VCDR values of 0.50 and 0.47, respectively [31, 35, 36]. Comparative cup-disc ratio findings using the Cirrus OCT are ACDR = 0.53 [20], VCDR = 0.51 [20], and 0.44 ± 0.18 [22]. The mean cup volume of 0.19 mm^3^ in our subjects is identical to the published data [20], and the rim area was 1.48 slightly higher than reported value of 1.32 by Cirrus OCT [20]. Gender however did not show any significant variation in ONH parameters which was consistent with other studies [20, 22]. Age was only associated with RA showing a negative correlation with age also consistent with what is reported in the literature [20, 24, 29, 37]. ACDR and VCDR did not show any significant correlation with age, contrary to that reported by Knight et al. [20]. Sex differences in optic disc parameters were statistically not signiﬁcant. Most previous studies have reported a lack of association of sex with optic disc parameters [38–40]. These differences in population normative values are most likely due to differences in the race, and discrepancies in OCT type are due to scanning patterns and optic disc and cup delineation algorithms between Stratus and Cirrus OCT [22].

### 4.2. Comparison between Normal and Glaucoma Patients

The Cirrus HD-OCT is seen in this study to have the ability to distinguish between healthy and Glaucomatous RNFL and ONH (Table 5). The mean (SD) average RNFL thickness for glaucoma subjects was 85.84 ± 13.11, showing a marked reduction from the normative average (SD) of 102.37 ± 7.45 *μ*m, about 16 microns reduction in RNFL. Average cup to disc ratio in glaucoma cases was 0.76 showing about a 50% increase in the normative value of 0.50. There was significant difference between RNFL and ONH parameters. The population mean RNFL layer is seen to reduce 11.4% in glaucoma cases. ONH parameters also showed similar differences between normal and glaucoma cases. There was an average increase of 50% in size of cup, larger cup volume, and a significantly reduced rim area, indicating ONH structural changes.

This research hoped to find out if some visual function parameters could predict RNFL changes. Stereoacuity demonstrated significant negative correlation with average RNFL (*p* < 0.05) and improved with increasing average RNFL thickness (Figure 3). Though a direct relationship between RNFL measured by OCT and stereoacuity has not yet been established, there are strong indications that stereoacuity is a function of a healthy retina. It is therefore a valid assumption that increasing RNFL fibres is associated with finer stereoacuity. Despite finding a significant correlation between stereoacuity and average RNFL in the present study, stereoacuity failed to predict correctly the average RNFL upon running linear regression. Contrast sensitivity showed weak positive correlation with nasal retinal nerve fibre layer quadrant (*p* < 0.001). No significant correlation was observed between RNFL quadrant thickness and measured visual function parameters. IOP was also shown to exhibit no correlation with ONH parameters, consistent with a report by Ruangvaravate and Neungton [41]. Spherical equivalent refractive error was observed to show significant negative correlation with disc area (*p*=0.02) which is consistent with a study by Leung et al. [30] Visual function parameters such as visual acuity, spherical equivalent refractive error. and IOP showed some correlation with RNFL parameters in the healthy study participants but failed to predict correctly RNFL and ONH variables.

Despite some correlation, visual function parameters failed to significantly predict RNFL and ONH parameters, which could be attributed to sample size used in this study, prompting further research to understand how RNFL and ONH changes affect visual functions such as contrast sensitivity and stereoacuity which could be an effective clinical tool in the early detection of glaucoma.

There are some limitations of this study that must be taken into consideration when generalising the findings. While the overall normative values were calculated from a population representative of the Ghanaian population, the sample used is relatively small and though sufficient to address the hypothesis of this study, it may not be large enough to be fully representative of the diverse ethnic background of the African population.

In addition, while care was taken to recruit the sample from the population some selection bias may be present because subjects' included in the study may be more likely to be those seeking eye care services. This may affect some parameters such as the prevalence of RE within the study groups. This may also limit the ability to generalize the study results to the overall populations, but it is likely reflective of those individuals seeking eye care services. Though no ethnic variation has been observed in studies on the prevalence of glaucoma in Ghana, the ethnic diversity of our study sample have predominance of the indigenous ethnic group, i.e., people from Accra. A multicentre population-based study involving subjects in the different regions of Ghana (representing the different tribal dominances) will provide more complete normative data and any ethnic differences that may exist in the normative values.

## 5. Conclusion

We have presented normative values for SD Cirrus HD-OCT 5000-measured RNFL thickness and ONH parameters in a healthy black Ghanaian population. The average RNFL thickness was 102.01 ± 7.45 *μ*m, thinner than that found for other African countries and significantly higher than that reported in other races using the Cirrus HD-OCT 5000 model. RNFL was thickest inferiorly, followed by superior quadrant, nasal quadrant, and temporal quadrant. Age predicted and correlate strongly with RNFL layers. Visual function parameters such as stereoacuity was associated with average RNFL. Spherical equivalent refractive error and visual acuity were seen to correlate with some RNFL and ONH parameters. Contrast sensitivity and stereoacuity should be added to the routine ocular examination procedure, as stereoacuity and contrast sensitivity have shown some correlation with RNFL changes. Findings may be of clinical value when assessing factors that influence these parameters and diagnosing glaucoma.

## Figures and Tables

**Table 1:** Distribution of clinical visual parameters among the nonglaucoma participants.

Variable	*N*	CS (triplets)			SA (sec arc)			IOP (mmHg)			LogMAR VA (MRSE)		
		Mean	95% CI	*p* value	Mean	95% CI	*p* value	Mean	95% CI	*p* value	Mean	95% CI	*p* value
Gender				0.693			0.427			0.549			0.399
Female	51	10.35 (1.16)	10.03–10.68		60.00 (97.49)	32.58–87.42		14.94 (2.45)	14.25–15.63		0.34 (0.33)	0.24–0.42	
Male	49	10.45 (1.26)	10.09–10.81		75.61 (98.31)	47.37–103.85		14.63 (2.68)	13.86–15.40		0.27 (0.35)	0.17–0.37	

Age				0.000^*∗*^			0.000^*∗*^			0.057			0.000^*∗*^
19–39	50	10.68 (1.04)	10.38–10.98		29.40 (15.14)	25.10–33.70		14.30 (2.45)	13.60–15.00		0.22 (0.30)	0.13–0.30	
40–59	35	10.49 (0.89)	10.18–10.79		61.71 (46.13)	45.87–77.56		14.94 (2.18)	14.19–15.69		0.26 (0.29)	0.15–0.35	
60+	15	9.27 (1.71)	8.32–10.21		209.00 (186.62)	105.66–312.34		16.07 (3.33)	14.22–17.91		0.71 (0.30)	0.5311–0.8650	

Refractive error				0.000^*∗*^			0.000^*∗*^			0.264			0.000^*∗*^
Plano	18	11.00 (0.77)	10.62–11.38		35.00 (26.23)	21.95–48.05		14.56 (2.50)	13.31–15.80		0.00 (0.00)	0.00–0.00	
Low hyperopia	25	10.48 (0.87)	10.12–10.84		44.40 (33.68)	30.50–58.30		14.44 (2.14)	13.56–15.32		0.25 (0.26)	0.14–0.35	
High hyperopia	5	8.40 (0.89)	7.29–9.51		231.00 (230.34)	−55.00–517.00		17.20 (1.09)	15.84–18.56		0.58 (0.38)	0.12–1.05	
Low myopia	38	10.55 (1.01)	10.22–10.88		43.82 (41.76)	30.09–57.54		14.71 (2.48)	13.89–15.53		0.24 (0.23)	0.16–0.31	
High myopia	14	9.79 (1.81)	8.74–10.83		157.50 (159.05)	65.67–249.33		15.07 (3.56)	13.01–17.13		0.88 (0.19)	0.77–0.98	
Total	100	10.40 (1.21)	10.16–10.64		67.65 (97.71)	48.26–87.04		14.79 (2.56)	14.28–15.30		0.30 (0.34)	0.24–0.37	

VA, visual acuity; IOP, intraocular pressure; CS, contrast sensitivity; SA, stereoacuity; MRSE, mean refractive spherical equivalent. ^a^One-way analysis of variance. ^*∗*^Significant.

**Table 2:** Mean (SD) RNFL values measured across normal subjects.

Variable	*N*	Mean (SD)	95% CI	*p*	Mean (SD)	95% CI	*p*	Mean (SD)	95% CI	*p*	Mean (SD)	95% CI	*p*	Mean (SD)	95% CI	*p*
		AVG_RNFL			SUP_RNFL			NSL_RNFL			TEMP_RNFL			INF_RNFL		
Age (years)				0.000^*∗*^			0.080			0.003^*∗*^			0.410			0.000^*∗*^
19–39	50	105.22 (7.10)	103.20–107.24		134.32 (14.69)	130.15–138.49		80.28 (11.50)	77.01–83.55		67.90 (9.36)	65.24–70.56		137.84 (13.79)	133.92–141.76	
40–59	35	101.54 (5.76)	99.56–103.52		130.43 (13.49)	125.79–135.06		75.09 (12.98)	70.63–79.54		66.40 (9.26)	63.22–69.58		132.69 (12.97)	128.23–137.14	
60+	15	94.80 (6.58)	91.16–98.44		124.87 (16.47)	115.75–133.99		68.73 (8.37)	64.10–73.37		64.27 (10.51)	58.45–70.08		121.60 (7.78)	117.29–125.91	

Gender				0.001^*∗*^			0.002^*∗*^			0.043^*∗*^			0.024^*∗*^			0.169
Female	51	104.40 (6.90)	102.90–106.78		135.86 (15.34)	131.55–140.18		79.16 (11.53)	75.91–82.40		68.92 (9.948)	66.12–71.72		135.47 (13.22)	131.75–139.19	
Male	49	99.80 (7.18)	97.73–101.86		127.04 (12.86)	123.35–130.74		74.20 (12.59)	70.59–77.82		64.65 (8.550)	62.20–67.11		131.65 (14.34)	127.53–135.77	

Refractive error				0.008^*∗*^			0.024^*∗*^			0.137			0.312			0.013^*∗*^
Plano	18	103.72 (7.71)	99.89–107.55		131.39 (15.69)	123.59–139.19		77.56 (11.08)	72.05–83.06		67.72 (8.923)	63.28–72.16		137.56 (12.38)	131.40–143.71	
Low hyperopia	25	103.20 (7.82)	99.97–106.43		133.32 (13.54)	127.73–138.91		80.08 (15.58)	73.65–86.51		64.96 (8.744)	61.35–68.57		134.44 (17.47)	127.23–141.65	
High hyperopia	5	94.00 (10.12)	81.43–106.57		122.00 (18.70)	98.79–145.21		68.80 (12.48)	53.31–84.29		61.40 (8.706)	50.59–72.21		122.20 (10.85)	108.73–135.67	
Low myopia	38	103.84 (6.19)	101.81–105.88		135.29 (14.22)	130.62–139.96		77.21 (9.31)	74.15–80.27		67.03 (10.612)	63.54–70.51		136.03 (11.71)	132.18–139.88	
High myopia	14	98.14 (5.92)	94.72–101.56		121.79 (11.72)	115.02–128.55		71.21 (12.67)	63.90–78.53		70.43 (7.949)	65.84–75.02		124.50 (9.45)	119.04–129.96	
Total	100	102.37 (7.45)	100.89–103.85		131.54 (14.79)	128.61–134.47		76.73 (12.26)	74.30–79.16		66.83 (9.488)	64.95–68.71		133.60 (13.84)	130.85–136.35	

RNFL, retinal nerve fibre layer; AVG, average; SUP, superior; NSL, nasal; TEMP, temporal; INF, inferior. ^a^One-way analysis of variance for subjects characteristics and RNFL values; *p*, *p* value. ^*∗*^Statistically significant.

**Table 3:** Mean (SD) ONH parameters measured across normal subjects.

Variable	*N*	RIM_AREA			DISC_AREA			VERT_CDR			AVG_CDR			CUP_VOLM		
		Mean (SD)	95% CI	*p*	Mean (SD)	95% CI	*p*	Mean (SD)	95% CI	*p*	Mean (SD)	95% CI	*p*	Mean (SD)	95% CI	*p*
Gender				0.368			0.391			0.826			0.752			0.200
Female	51	1.50 (0.22)	1.44–1.57		2.11 (0.39)	2.00–2.22		0.47 (0.13)	0.43–0.51		0.49 (0.14)	0.46–0.54		0.21 (0.16)	0.16–0.25	
Male	49	1.47 (0.19)	10.41–10.52		2.04 (0.41)	1.92–2.16		47 (0.12)	0.43–0.50		0.49 (0.11)	0.46–0.53		0.17 (0.13)	0.13–0.20	
Total	100	1.48 (0.21)	1.44–1.53		2.08 (0.40)	1.99–2.16		47 (0.13)	0.45–49		0.49 (0.13)	0.47–0.52		0.19 (0.14)	0.16–0.22	

Age				0.004^*∗*^			0.010^*∗*^			0.425			0.636			0.076
19–39	50	1.55 (0.19)	1.49–1.61		2.19 (0.41)	2.07–2.30		0.46 (0.14)	0.42–0.49		0.49 (0.15)	0.45–0.53		0.19 (0.15)	0.15–0.23	
40–59	35	1.43 (0.21)	1.36–1.50		2.01 (0.41)	1.87–2.15		0.49 (0.11)	0.46–0.53		0.51 (0.11)	0.47–0.55		0.21 (0.15)	0.17–0.27	
60+	15	1.39 (0.19)	1.28–1.49		1.86 (0.25)	1.72–2.00		0.47 (0.10)	0.41–0.52		0.47 (0.11)	0.41–0.53		0.11 (0.09)	0.07–0.16	
Total	50	1.48 (0.21)	1.44–1.53		2.08 (0.40)	1.99–2.16		47 (0.13)	0.45–0.49		0.49 (0.13)	0.47–0.52		0.19 (0.14)	0.16–0.22	

Refractive error				0.399			0.017			0.316			0.125			0.135
Plano	18	1.55 (0.19)	1.45–1.64		2.3 (0.38)	2.12–2.50		0.49 (0.13)	0.43–0.56		0.54 (0.13)	0.47–0.59		0.22 (0.15)	0.15–0.30	
Low hyperopia	25	1.47 (0.22)	1.38–1.56		2.06 (0.39)	1.90–2.22		0.49 (0.09)	0.45–0.53		0.52 (0.09)	0.48–0.55		0.21 (0.17)	0.14–0.29	
High hyperopia	5	1.54 (0.28)	1.19–1.89		1.99 (0.25)	1.69–2.31		0.43 (0.10)	0.30–0.56		0.44 (0.09)	0.32–0.57		0.09 (0.08)	−0.01–0.19	
Low myopia	38	1.49 (0.21)	1.42–1.56		2.08 (0.41)	1.94–2.21		0.47 (0.14)	0.43–0.52		0.49 (0.14)	0.45–0.54		0.19 (0.13)	0.14–0.23	
High myopia	14	1.41 (0.17)	1.31–1.50		1.83 (0.35)	1.62–2.04		0.41 (0.14)	0.33–0.49		0.43 (0.15)	0.35–0.51		0.12 (0.11)	0.06–0.19	
Total	100	1.48 (0.21)	1.44–1.52		2.08 (0.40)	1.99–2.16		0.47 (0.13)	0.45–0.49		0.49 (0.13)	0.47–0.52		0.19 (0.14)	0.16–0.22	

VERT CDR, vertical cup-disc ratio; AVG CDR, average cup-disc ratio; CUP VOLM, cup volume. ^a^One-way analysis of variance for subjects characteristics and ONH parameters; *p*, *p* value. ^*∗*^Statistically significant.

**Table 4:** Bivariate correlationsa between clinical variables, optic nerve head, and RNFL parameters.

Variable	*M* (SD)	AVG RNFL *R* (*p*)	SUP_RNFL *R* (*p*)	NSL_RNFL *R* (*p*)	TEMP_RNFL *R* (*p*)	INF_RNFL *R* (*p*)
Gender	—	−0.340 (0.000^*∗*^)	−0.274 (0.001^*∗*^)	−0.312 (0.000^*∗*^)	−0.143 (0.17)	−0.401 (0.000^*∗*^)
Age	40.68 (14.48)	−0.443 (000^*∗*^)	−0.239^*∗*^ (0.017^*∗*^)	−0.263 (0.008^*∗*^)	−0.176 (0.079)	−0.407^*∗∗*^ (0.000^*∗*^)
RE	−0.45 (1.53)	0.073 (0.502)	0.172 (0.214)	0.123 (0.244)	−0.234 (0.021)	0.112 (0.282)
CS	10.40 (1.21)	0.544 (0.061)	0.000 (0.998)	0.221^*∗*^ (0.021^*∗*^)	0.051 (0.611)	0.066 (0.512)
SA	67.65 (97.71)	−0.265 (0.008^*∗*^)	−0.130 (0.199)	−0.250^*∗*^ (0.012^*∗*^)	−0.054 (0.596)	−0.148 (0.143)
IOP	14.79 (2.56)	−0.143 (0.156)	−0.020 (0.842)	0.118 (0.244)	−0.110 (0.275)	−0.185 (0.065)
RIM_AREA	1.48 (0.21)	0.310 (0.002^*∗*^)	0.270^*∗∗*^ (0.007^*∗*^)	−0.008 (0.936)	−0.024 (0.809)	0.288^*∗∗*^ (0.004^*∗*^)
DISC_AREA	2.08 (0.40)	0.441^*∗∗*^ (0.000^*∗*^)	0.332^*∗∗*^ (0.001^*∗*^)	0.133 (0.188)	0.068 (0.503)	0.390^*∗∗*^ (0.000^*∗*^)
VERT_CDR	0.47 (0.13)	0.110 (0.275)	0.066 (0.511)	0.168 (0.095)	0.037 (0.717)	0.081 (0.425)
AVG_CDR	0.49 (0.13)	0.200 (0.046)	0.119 (0.240)	0.205^*∗*^ (0.041^*∗*^)	0.047 (0.646)	0.172 (0.087)
CUP_VOLM	0.19 (0.14)	0.198 (0.048^*∗*^)	0.084 (0.406)	0.285^*∗∗*^ (0.004^*∗*^)	0.029 (0.776)	0.153 (0.128)

RNFL, retinal nerve fibre layer; RE, spherical equivalent refractive error; AVG, average; SUP, superior; NSL, nasal; TEMP, temporal; INF, inferior; VERT CDR, vertical cup-disc ratio; AVG CDR, average cup-disc ratio; CUP VOLM, cup volume. ^a^Pearson correlation coefficient where *r*^2^ ≥ 0.05 (5%). ^*∗*^Statistically significant.

**Table 5:** Comparisons of RNFL and ONH parameters of the normal and glaucoma patients across gender and age.

Variable	Gender							
	Female		Male		Total			
	Normal *M* (SD)	Glaucoma *M* (SD)	Normal *M* (SD)	Glaucoma *M* (SD)	Normal *M* (SD)	Glaucoma *M* (SD)	*F*	*p* value
Age	36.80 (13.11)	59.64 (14.38)	44.71 (14.86)	59.46 (13.82)	40.68 (14.48)	59.54 (14.04)	128.46	<0.001
AVG_RNFL	104.84 (6.90)	91.51 (13.54)	99.80 (7.18)	90.15 (15.29)	102.37 (7.45)	90.74 (14.55)	58.03	<0.001
SUP_RNFL	135.86 (15.34)	112.19 (24.03)	127.04 (12.86)	109.77 (26.24)	131.54 (14.79)	110.81 (25.29)	59.41	<0.001
INF_RNFL	135.47 (13.22)	113.55 (26.16)	131.65 (14.34)	109.55 (27.45)	133.60 (13.84)	111.27 (26.93)	62.42	<0.001
TEMP_RNFL	68.92 (9.95)	58.34 (13.18)	64.65 (8.55)	58.45 (13.78)	66.83 (9.49)	58.40 (13.50)	32.77	<0.001
NSL_RNFL	79.16 (11.53)	70.69 (12.59)	74.20 (12.59)	71.89 (12.31)	76.73 (12.26)	71.38 (12.42)	13.56	<0.001
RIM_AREA	1.50 (0.22)	1.22 (0.28)	1.47 (0.20)	1.16 (0.31)	1.48 (0.21)	1.18 (0.30)	86.26	<0.001
DISC_AREA	2.11 (0.40)	2.51 (0.50)	2.04 (0.41)	2.44 (0.52)	2.08 (0.40)	2.47 (0.51)	48.14	<0.001
VERT_CDR	0.47 (0.13)	0.71 (0.08)	0.47 (0.12)	0.73 (0.09)	0.47 (0.13)	0.72 (0.09)	453.66	<0.001
AVG_CDR	0.50 (0.14)	0.74 (0.07)	0.49 (0.12)	0.75 (0.08)	0.50 (0.13)	0.75 (0.08)	511.99	<0.001
CUP_VOLM	0.21 (0.16)	0.62 (0.30)	0.17 (0.13)	0.73 (0.36)	0.19 (0.14)	0.68 (0.34)	200.27	<0.001

RNFL, retinal nerve fibre layer; RE, spherical equivalent refractive error; AVG, average; SUP, superior; NSL, nasal; TEMP, temporal; INF, inferior; VERT CDR, vertical cup-disc ratio; AVG CDR, average cup-disc ratio; CUP VOLM, cup volume.

**Table 6:** Comparison of RNFL and OHN parameters^a^ with published normative data.

Research	OCT type	Country/race	*N*	Mean age (range)	ARNFL	SRNFL	IRNFL	NRNFL	TRNFL	ACDR	VCDR	RA	DA	CV
Current study	Cirrus SD HD 5000	Ghana/African	100	40.6 (20–78)	102.4 ± 7.5	131.5 ± 14.8	133.6 ± 13.3	76.7 ± 12.2	66.8 ± 9.5	0.5 ± 0.1	0.5 ± 0.1	1.5 ± 0.2	2.0 ± 0.4	0.2 ± 0.1
Manassakorn et al. [26]	Stratus SD	United States	42	57.9 (40–78)	98.2 ± 10.8	122.8	127.4	73.6	69.4	0.6	0.5	1.6	2.3	
Budenz et al. [21]	Stratus TD	USA	328	47.4 (18–85)	100.1 ± 11.6	124.2 ± 17.9	126.1 ± 17.8	80.9 ± 18.1	69.0 ± 12.7					
Bendschneider et al. [27]	Spectralis SD	Germany	170	(20–78)	97.2 ± 9.7	118.0 ± 14.5	123.7 ± 16.4	76.4 ± 15.0	68.8 ± 11.1				2.2 ± 0.5	
Hirasawa et al. [28]	Topcon SD	Japan	251	(2070+)	101.9 ± 8.4	123.9 ± 13.6	125.5 ± 13.1	79.6 ± 13.6	78.6 ± 13.3					
Knight et al. [20]	Cirrus SD	Mixed	271	46.2 (18–84)	94.0 ± 0.6	119.0 ± 0.9	123.2 ± 1.0	69.8 ± 0.7	64.0 ± 0.6	0.47 ± 0.01	0.45 ± 0.01	0.47 ± 0.01	1.81 ± 0.02	0.15 ± 0.01
Knight et al.^*∗*^ [20]	Cirrus HD	African decent	51/284	45.8 (18–84)	93.9 ± 1.2	119.6 ± 1.9	125.2 ± 2.0	73.0 ± 1.5	57.8 ± 1.3	0.53 ± 0.02	0.51 ± 0.02	1.32 ± 0.02	1.93 ± 0.05	0.20 ± 0.02
Tariq et al. [22]	Cirrus SD	Australia	1521	17.3 (16–19)	99.4 ± 9.7	124.8 ± 15.9	128.5 ± 17.2	74.3 ± 12.6	69.8 ± 11.5	0.4 ± 0.2	0.4 ± 0.2	1.5 ± 0.3	2.0 ± 0.4	0.1 ± 0.1
Appukuttan et al. [19]	Spectralis SD	India	105	(20–75)	101.4 ± 8.6	125.3 ± 13.7	128.3 ± 14.7	72.0 ± 12.1	79.7 ± 7.7					
Sani et al. [25]	Stratus SD	Nigerian	110	30.5 (18–51)	104.2 ± 10.7	135.3 ± 20.4	129.2 ± 16.9	85.1 ± 23.6	67.2 ± 13.2					
Mashige and Oduntan [24]	iVue-SD	South Africa	600	28.2 (10–66)	110.0 ± 7.4	132.0 ± 10.5	135.0 ± 9.7	87.2 ± 13.2	73.6 ± 15.6					

^a^Mean and standard deviation (SD) for optic nerve head and optic nerve head parameters. RNFL, retinal nerve fibre layer; A, average; S, superior; N, nasal; T, temporal; I, inferior; RA, rim area; DA, disc area; VCDR, vertical cup-disc ratio; ACDR, average cup-disc ratio; CV, cup volume. ^*∗*^Cirrus data (adjusted values for African American race).

## Data Availability

The data used to support the findings of this study are available from the corresponding author upon request.

## Supplementary Materials

Supplementary Materials Supplementary Table 1: univariate analysis of variance for factors significant for RNFL thickness. A generalised linear model univariate analysis of variance for demographic and clinical factors significant for RNFL thickness showed that only age (*p*=0.006) and sex (*p*=0.017) had significant effect on average RNFL thickness. Supplementary Table 2: parameter estimates showing the effect of each predictor on RNFL thickness. The parameter estimates for RNFL thickness show an increase of 4.42 *μ*m for those aged 19–30 and 2.67 *μ*m increase for those aged 40–59 over those 60+ years per yearly decrease in age. Females showed 5.5 *μ*m increase in average RNFL thickness compared with males. Supplementary Table 3: binary logistic regression for glaucoma and normal patients adjusted for age. Binary logistic regression analysis with age correction revealed that a yearly increase in age increases the likelihood of getting glaucoma by 1.19 times.
