# Supplementary material for: Normative Values of Retinal Nerve Fibre Layer Thickness and Optic Nerve Head Parameters and Their Association with Visual Function in an African Population
Source: J Ophthalmol. 2020 Feb 11;2020:7150673. doi: 10.1155/2020/7150673 (PMC7036135; doi:10.1155/2020/7150673)
Supplement: Supplementary Materials — Supplementary Table 1: univariate analysis of variance for factors significant for RNFL thickness. A generalised linear model univariate analysis of variance for demographic and clinical factors significant for RNFL thickness showed that only age (p=0.006) and sex (p=0.017) had significant effect on average RNFL thickness. Supplementary Table 2: parameter estimates showing the effect of each predictor on RNFL thickness. The parameter estimates for RNFL thickness show an increase of 4.42 μm for those aged 19–30 and 2.67 μm increase for those aged 40–59 over those 60+ years per yearly decrease in age. Females showed 5.5 μm increase in average RNFL thickness compared with males. Supplementary Table 3: binary logistic regression for glaucoma and normal patients adjusted for age. Binary logistic regression analysis with age correction revealed that a yearly increase in age increases the likelihood of getting glaucoma by 1.19 times. [file 7150673.f1.pdf]

SUPPLEMENTARY TABLES

Table 1 GLM Univariate analysis of variance for Factors significant for RNFL thickness

Tests of Between-Subjects Effects															
Dependent Variable:	AVG RNFL			SUP RNFL			NSL RNFL			INF RNFL			TEMP RNFL		
Source	Type III Sum of Squares	F	Sig.	Type III Sum of Squares	F	Sig.	Type III Sum of Squares	F	Sig.	Type III Sum of Squares	F	Sig.	Type III Sum of Squares	F	Sig.
Corrected Model	2265.598[a]	2.457	0.002	6708.984[a]	1.572	0.076	2147.254[a]	3.173	0.011	5532.238[a]	1.441	0.123	2595.331[a]	1.438	0.124
Intercept	542857.02	12950.35	0	903182.45	4655	0	423167.71	3126.09	0	909793.35	5212.45	0	234369.83	2856.911	0
Age	460.16	5.49	0.006	112.17	0.29	0.75	1428.24	5.28	0.007	537.29	1.54	0.221	354.731	2.162	0.122
Refractive error	202.69	1.21	0.314	1497.87	1.93	0.114				455.16	0.65	0.627	773.459	2.357	0.061
Gender	251.73	6.01	0.017	1053.12	5.43	0.022	312.66	2.31	0.132	51.98	0.3	0.587	499.463	6.088	0.016
Age * Refractive error	249.64	0.99	0.436	1073.05	0.92	0.484				488.15	0.47	0.831	661.634	1.344	0.248
Age * Gender	15.03	0.18	0.836	384.75	0.99	0.376	128.83	0.48	0.623	99.45	0.28	0.753	177.786	1.084	0.343
Refractive error * Gender	28.19	0.17	0.954	525.6	0.68	0.61				693.13	0.99	0.417	305.31	0.93	0.451
Age * Refractive error * Gender	18.95	0.15	0.929	601.66	1.03	0.383				452.89	0.86	0.463	560.144	2.276	0.086
Error	3227.71			14939.86			12724.46			13439.76			6316.779		
Total	1053455			1751926			603621			1803868			455537		
Corrected Total	5493.31			21648.84			14871.71			18972			8912.11		
	a. R Squared = .412 (Adjusted R Squared = .245)			a. R Squared = .310 (Adjusted R Squared = .113)			a. R Squared = .144 (Adjusted R Squared = .099)			a. R Squared = .292 (Adjusted R Squared = .089)			a. R Squared = .291 (Adjusted R Squared = .089)		

Abbreviations: RNFL, retinal nerve fibre layer; RE, spherical equivalent refractive error; AVG, average; SUP, superior; NSL, nasal; TEMP, temporal; INF, inferior;
VERT CDR, vertical cup-disc ratio; AVG CDR, average cup-disc ratio; CUP VOLM, cup volume

Table 2 Parameter estimates for RNFL parameter

Dependent Variable:	AVG RNFL				SUP RNFL				NSL RNFL				INF RNFL				TEMP INFL			
**Parameter**	B	t	Sig.	95% CI	B	t	Sig.	95% CI	B	t	Sig.	95% CI	B	t	Sig.	95% CI	B	t	Sig.	95% CI
**Intercept**	94	32.46	0	88.23-99.77	118	18.94	0	105.60-130.40	67.11	17.3	0	59.41-74.81	119.4	20.21	0	107.63-131.17	71.200	17.578	.000	63.134-79.266
**19 - 39**	4.42	0.44	0.662	-15.63-24.46	15.47	0.71	0.477	-27.65-58.59	12.24	2.62	0.01	2.97-21.51	-20.45	-1	0.323	-81.8	11.300	.803	.425	-16.737-39.337
**40 - 59**	2.67	0.56	0.574	-6.75-12.08	-4.67	-0.46	0.648	-24.92-15.59	5.14	1.1	0.274	-4.13-14.41	0.93	0.1	0.923	-38.43	-4.200	-.635	.527	-17.371-8.971
**60 +**	0[a]				0[a]				0[a]				0[a]				0[a]			
**Plano**	0	0	1	-10.53-10.53	0.67	0.06	0.953	-21.98-23.31	-	-	-	-	15.33	1.42	0.159	-42.96	-6.000	-.811	.420	-20.726-8.726
**Low Hyperopia**	-4	-0.56	0.574	-18.12-10.12	-2	-0.13	0.896	-32.38-28.38	-	-	-	-	-7.4	-0.51	0.611	-57.64	-18.200	-1.834	.070	-37.957-1.557
**High Hyperopia**	-9	-1.66	0.101	-19.79-1.79	-8.5	-0.73	0.468	-31.71-14.71	-	-	-	-	-5.9	-0.53	0.595	-44.02	-17.200	-2.270	.026	-32.290--2.110
**Low Myopia**	5	0.7	0.483	-9.12-19.12	15	0.98	0.329	-15.38-45.38	-	-	-	-	15.6	1.08	0.284	-57.64	-14.200	-1.431	.156	-33.957-5.557
**High Myopia**	0[a]				0[a]			-27.65-58.59	-				0[a]				0[a]			
**Female**	5.5	1.02	0.313	-5.29-16.29	10.5	0.9	0.37	-24.92-15.59	4.06	0.66	0.51	-8.12-16.23	10.6	0.96	0.341	-44.02	-1.700	-.224	.823	-16.790-13.390
**Male**	0[a]				0[a]				0[a]				0[a]				0[a]			

Abbreviations: RNFL, retinal nerve fibre layer; RE, spherical equivalent refractive error; AVG, average; SUP, superior; NSL, nasal; TEMP, temporal; INF, inferior;
VERT CDR, vertical cup-disc ratio; AVG CDR, average cup-disc ratio; CUP VOLM, cup volume

Table 3 Binary Logistic Regression: Glaucoma and Normals Adjusted for age

	B	S.E.	Wald	df	Sig.	Exp(B)	95% C.I.for EXP(B)	
							Lower	Upper
AGE	.177	.053	11.356	1	.001	1.19	1.08	1.32
AVG_RNFL	.412	.220	3.491	1	.062	1.51	0.98	2.32
SUP_RNFL	-.099	.060	2.766	1	.096	0.91	0.81	1.02
NSL_RNFL	-.161	.062	6.660	1	.010	0.85	0.75	0.96
TEMP_RNFL	-.118	.075	2.490	1	.115	0.89	0.77	1.03
INF_RNFL	-.122	.067	3.376	1	.066	0.88	0.78	1.01
RIM_AREA	9.061	3.239	7.826	1	.005	8610.65	15.07	4.92E+6
DISC_AREA	-4.056	1.796	5.101	1	.024	0.02	5.13E-4	0.59
VERT_CDR	54.764	19.578	7.825	1	.005	6.08E+23	1.312E+7	2.81E+40
AVG_CDR	49.447	14.932	10.965	1	.001	2.98E+21	5.801E+8	15.3E+34
CUP_VOLM	-2.513	3.777	.443	1	.506	0.08	4.94E-4	132.77
Constant	-66.998	18.586	12.995	1	.000	0.00		

Abbreviations: RNFL, retinal nerve fibre layer; RE, spherical equivalent refractive error; AVG, average; SUP, superior; NSL, nasal; TEMP, temporal; INF, inferior; VERT CDR, vertical cup-disc ratio; AVG CDR, average cup-disc ratio; CUP VOLM, cup volume
